# Advancing quantum imaging: Electrical tunability enabled by versatile liquid crystals

**DOI:** 10.1126/sciadv.adz8962

**Published:** 2026-01-28

**Authors:** Dong Zhu, Shi-Hui Ding, Rui Sun, Zhi-Xiang Li, Wen Chen, Yi-Heng Zhang, Si-Jia Liu, Yi-Ming Wang, Fang-Tianyu Chen, Peng Chen, Yan-Qing Lu

**Affiliations:** National Laboratory of Solid State Microstructures, Key Laboratory of Intelligent Optical Sensing and Manipulation, College of Engineering and Applied Sciences, and Collaborative Innovation Center of Advanced Microstructures, Nanjing University, Nanjing 210093, China.

## Abstract

Optical image processing techniques like edge detection demonstrate unique superiority in various applications. Synergistically combined with heralded quantum imaging, they have notable potential for identifying light-sensitive samples under minimal illumination. However, present schemes usually lack the capability to control the quantum imaging function dynamically. Here, we introduce versatile liquid crystals to realize heralded single-photon imaging and electrically tunable multimode switching. On the basis of the bichiral cholesteric liquid crystal modulator, different morphology information of the target can be extracted with a high signal-to-noise ratio, including the entire shape and its fine outline. The nematic liquid crystal, leveraging its electrically tunable properties, is explored to select specific polarizations of heralded single photons, enabling dynamic remote switching in trimode quantum imaging. To further improve the time efficiency, an ultrafast remote switching is presented on the basis of the appealing ferroelectric liquid crystals. This work supplies a unique imaging platform for photon-limited scenarios and reveals the unprecedented possibilities of soft matters in quantum information processing.

## Teaser

Versatile liquid crystals enable the multimode heralded single-photon imaging with an electrically tunable remote switch.

## INTRODUCTION

Nowadays, optical information processing attains growing attention benefiting from its high efficiency and high-speed parallel capacity (*1*). Compared to conventional electronics, optical processing can avoid extra energy loss and mitigate deformation and degradation of any components induced by the Joule effect. Recently, it has witnessed sustained demands in machine vision (*2*), autonomous vehicles (*3*), and microscopy (*4*). In particular, optical edge detection can extract the fine morphology information of the target object, drastically decreasing the handled data quantity, and thus can accelerate and improve the subsequent sections of image processing (*5*). One typical method is to establish the appropriate transfer function of an imaging system (*6*–*9*), which usually demonstrates a linear form in the spatial frequency domain, acting as a differentiation operation on the target image. Another way is to produce the optical differential interference induced by the lateral shear (*10*–*12*), so that the edge and variation of an image can be highlighted. In addition, the phase contrast method can also be used with spatial filter (*13*–*16*), like spiral phase contrast imaging, which can show the two-dimensional outline of a sample without noticeable energy loss. In practical applications, the selection of those optical methods can be dictated by specific task requirements.

However, in some photon-limited scenarios, especially the recognition of light-sensitive samples, detecting low-light illumination across the full scene is highly required, which always suffers from the ambient noise and demonstrates a very low signal-to-noise ratio (SNR). To address this issue, quantum entangled source featuring nonclassical correlations has been introduced recently (*17*–*25*). The high-sensitivity camera, combined with quantum mechanics, is triggered by heralded photons. Heralded single-photon imaging with coincidence measurement reveals a great robustness against the background and sensor noise, exhibiting a much higher SNR beyond classical cases (*26*), whereas most quantum imaging schemes lack the capability of dynamically manipulating entangled photons, resulting in fixed and limited functionality. In addition, both the entire morphology and the fine outline of an object are simultaneously required in corresponding applications (*27*–*32*), e.g., living photodynamic bacteria microscopy. As a high-performance and dynamic-tunable platform, liquid crystal (LC) (*33*–*36*) demonstrates intriguing merits of molecular self-organization, versatile sensitive stimuli-responsiveness, and multidimensional light manipulation. However, thus far, traditional LC devices have been extremely confined to the manipulation of classical light (*37*–*39*). Leveraging the unique nonlocality of entangled photons to explore the abundant resources offered by versatile LCs remains to be a substantial challenge in quantum imaging.

In this work, we propose an electrically tunable scheme of heralded single-photon imaging based on polarization-entangled source and versatile LCs, including cholesteric LCs (CLCs), nematic LCs (NLCs), and ferroelectric LCs (FLCs). The imaging device is composed of two opposite-handed CLC layers encoded with different orientations. Such a bichiral CLC device demonstrates two circular polarization–selective reflected modes, contributing to the bright-field imaging or edge detection. Polarization-entangled photons are generated by using a polarization Sagnac interferometer. For the remote switching in the heralding arm, when one of the heralding photons’ polarization is selected out, the corresponding imaging mode can be simultaneously selected under coincidence measurement. Therein, an NLC wave plate is applied to enable different polarization selection based on its electrically tunable effect. Trimode imaging switching can be realized, including bright-field imaging, edge detection, and their superposition. For all quantum imaging schemes based on different LCs, the noise can be drastically suppressed to obtain a high SNR. To further improve the time efficiency, a fast-response FLC wave plate is demonstrated to implement ultrafast dual-mode switching. This dynamic quantum imaging scheme explores more opportunities for versatile LCs in the emerging frontiers of quantum information processing and optical computing.

## RESULTS

### Principle of electrically tunable heralded single-photon imaging

To derive heralded single-photon imaging, the polarization-entangled source (*40*) is first prepared in the form of Bell state and can be expressed as $|\psi_{1}\rangle =\frac{1}{\sqrt{2}}\left({|H\rangle }_{i}{|V\rangle }_{s}+{|V\rangle }_{i}{|H\rangle }_{s}\right)=\frac{1}{\sqrt{2}i}\left({|L\rangle }_{i}{|L\rangle }_{s}-{|R\rangle }_{i}{|R\rangle }_{s}\right)$. The signal and idler photons within an entangled photon pair exhibit a pronounced time correlation. The intensified charge-coupled device (ICCD) camera will register signal photons exclusively within a nanosecond temporal window synchronized with idler photon detection. This coincidence measurement leverages the time-energy entanglement. It ensures that the temporal windows containing signal photons are systematically identified and retained, whereas intervals exhibiting predominant noise accumulation are deliberately excluded. Thus, the heralded single-photon imaging demonstrates superior denoising capability and exhibits a much higher SNR than classical cases acquired under the same exposure time.

**Fig. 1.**
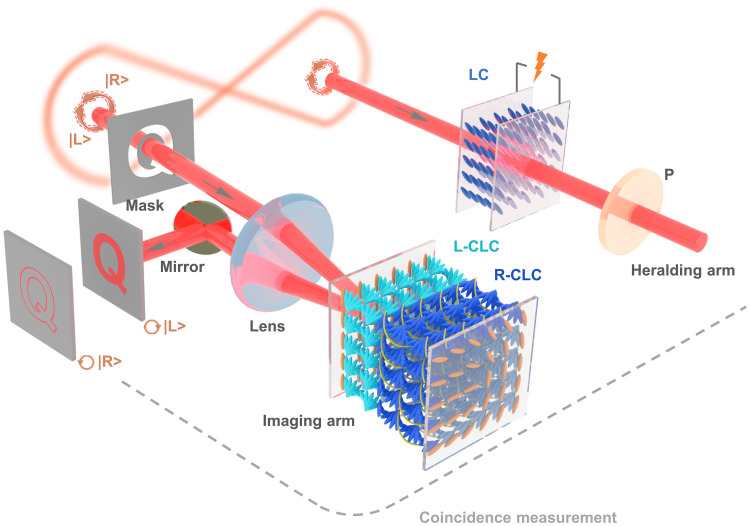
Schematic illustration of the electrically tunable heralded single-photon imaging based on versatile LCs. The imaging arm is illuminated by the signal photons and operates in the bright-field or edge detection imaging mode based on the manipulation of the bichiral CLC device. The polarization state of the idler photon is selected out by the combination of an LC wave plate and a polarizer and acts as a trigger on the ICCD camera. The selection can be dynamically adjusted on demand by the external voltage and the corresponding imaging modes will be remotely switched under the coincidence measurement. The gray arrows indicate the propagation direction of entangled photons. The gray dashed line stands for the electrical path. In the imaging arm, light blue and dark blue rods in the LC device represent the L-CLC and R-CLC, respectively. Yellow springs surrounding the R-CLC represent the polymer scaffold. The orange rods at the substrates indicate the LC director’s orientation distribution at the surface.

A high-efficiency modulator is required in our quantum imaging design to facilitate high-quality image acquisition and the execution of diverse processing tasks. A liquid crystalline mesophase with chirality, namely, CLC, exhibits great potential in optical field control. Derived from its periodically helically twisted superstructure, a spin-determined Bragg reflection is exhibited together with the unique geometric phase modulation (*41*–*45*). The circularly polarized component matched the CLC handedness can be reflected and endowed with designed geometric phase of ±2α within the photonic bandgap (PBG) from *n*_o_*p* to *n*_e_*p*. The sign (±) depends on the chirality of polarization, and the *n*_o_/*n*_e_ is the ordinary/extraordinary refractive index, and *p* is CLC’s helical pitch. Here, a single LC sample is fabricated to incorporate two CLC layers with opposite handedness but an identical PBG. In this case, the left circularly polarized (LCP) and right circularly polarized (RCP) components are deflected at different interfaces, respectively, and obtain spin-decoupled geometric phases (*46*). This bichiral CLC performs as a high-efficiency and spin-decoupled reflective planar modulator within its PBG, featuring two polarization-dependent reflective channels (Fig. 1). In addition, this manipulation will not result in obvious decoherence between two circular polarization channels, and its modulation function can be expressed as

$$a|L\rangle +b|R\rangle \to {\mathrm{ae}}^{i\Phi_{L}}|L\rangle +{\mathrm{be}}^{i\Phi_{R}}|R\rangle$$ (1)

The parameters *a* and *b* are determined by the polarization state of incident light. Φ_L_ and Φ_R_ represent the induced phase for LCP and RCP components, respectively. These phase components depend on the surface orientation distribution. The detailed analysis of such a bichiral CLC device can be seen in Supplementary Text 1. If the device is flipped, it remains unchanged for RCP incidence, whereas the induced phase modulation for the LCP will turn to the conjugated phase of the RCP, i.e., Φ_L_ = −Φ_R_.

Here, considering the incidence from the left-handed CLC (L-CLC) layer, a spiral phase profile and a uniform pattern are encoded into right-handed CLC (R-CLC) and L-CLC layers, respectively, to realize dual-mode imaging via opposite circular polarization channels. For the RCP channel, a radial Hilbert transform is performed in a 4*f* system to enhance the edge isotropically based on the spiral phase contrast method (*13*). The corresponding modulation effect can be approximatively seen as the Wirtinger derivative, i.e., $\propto \frac{\partial }{\partial x}\pm i\frac{\partial }{\partial y}$. For the LCP channel, the bright-field imaging is implemented to demonstrate the morphology information of the target. In all, the modulation of CLC imaging device can be written as $M_{s}={|R,1\rangle }_{s}{\langle R|}_{s}+{|L,0\rangle }_{s}{\langle L|}_{s}$. Here, ${|R,1\rangle }_{s}$ and ${|L,0\rangle }_{s}$ represent the state of signal photons in the edge-enhancement mode and bright-field imaging mode with the corresponding circular polarization, respectively. The entangled photon source is used to illuminate the target and the proposed bichiral CLC imaging device. After the transformation, the state of the entangled photon pair can be written as

$$|\psi_{2}\rangle =\left(I_{i}⊗M_{s}\right)|\psi_{1}\rangle =\frac{1}{\sqrt{2}i}\left({|L\rangle }_{i}{|L,0\rangle }_{s}-{|R\rangle }_{i}{|R,1\rangle }_{s}\right)$$ (2)

Here, the operator *I_i_* is the identity matrix.

Besides, polarization entanglement (*47*, *48*) provides a remote control scheme under the coincidence measurement. Selective measurement of the idler photon’s polarization state induces the polarization state collapse of the signal photon. The LC wave plate with unique electro-optical effect can serve as an electrically tunable relay switch. Combined with a polarizer, the corresponding polarization-selection matrix can be expressed as

$$\begin{array}{c} S_{i}=\frac{1}{2}\left[\left(\text{cos}\frac{\Delta \Gamma }{2}+i\text{sin}\frac{\Delta \Gamma }{2}e^{-i2\theta }\right){|L\rangle }_{i}{\langle L|}_{i}\right.\\ \left.-\left(\text{cos}\frac{\Delta \Gamma }{2}+i\text{sin}\frac{\Delta \Gamma }{2}e^{i2\theta }\right){|L\rangle }_{i}{\langle R|}_{i}\right]\\ \left.-\left(\text{cos}\frac{\Delta \Gamma }{2}+i\text{sin}\frac{\Delta \Gamma }{2}e^{-i2\theta }\right){|R\rangle }_{i}{\langle L|}_{i}\right]\\ \left.+\left(\text{cos}\frac{\Delta \Gamma }{2}+i\text{sin}\frac{\Delta \Gamma }{2}e^{i2\theta }\right){|R\rangle }_{i}{\langle R|}_{i}\right] \end{array}$$ (3)

The ΔΓ and θ demonstrate the phase retardation and orientation angle of an equivalent LC wave plate, respectively. In some cases, an additional wave plate is inserted, and the polarization-selection matrix can also be equivalently simplified to a form similar to Eq. 3. In versatile liquid crystalline mesophases, these parameters can be respectively adjusted by an external electric field. For example, we can apply an appropriate voltage to select the RCP/LCP component of the idler photon, and the output state of the signal photon is collapsed into

$$\rho_{s}=\frac{{\text{Tr}}_{i}\left[\left(S_{i}⊗M_{s}\right)|\psi_{1}\rangle \langle \psi_{1}|{\left(S_{i}⊗M_{s}\right)}^{†}\right]}{\text{Tr}\left[\left(S_{i}⊗M_{s}\right)|\psi_{1}\rangle \langle \psi_{1}|{\left(S_{i}⊗M_{s}\right)}^{†}\right]}={|R,1\rangle }_{s}{\langle R,1|}_{s}$$ (4)

$$\rho_{s}=\frac{{\text{Tr}}_{i}\left[\left(S_{i}⊗M_{s}\right)|\psi_{1}\rangle \langle \psi_{1}|{\left(S_{i}⊗M_{s}\right)}^{†}\right]}{\text{Tr}\left[\left(S_{i}⊗M_{s}\right)|\psi_{1}\rangle \langle \psi_{1}|{\left(S_{i}⊗M_{s}\right)}^{†}\right]}={|L,0\rangle }_{s}{\langle L,0|}_{s}$$ (5)

representing the edge detection and bright-field imaging, respectively. In particular, for the vertical polarizer operator ${|V\rangle }_{i}{\langle V|}_{i}$, the state of signal photons is collapsed into $\rho_{s}=\frac{1}{2}\left({|L,0\rangle }_{s}+{|R,1\rangle }_{s}\right)\left({\langle L,0|}_{s}+{\langle R,1|}_{s}\right)$, which corresponds to a superposition image. Therefore, on the basis of the electrically tunable and nonlocally positioned switching, the polarization-dependent quantum imaging mode can be dynamically selected without any change in the imaging arm. This control strategy leverages the flexibility provided by the heralding arm.

### Experimental scheme of heralded single-photon imaging based on the bichiral CLC device

**Fig. 2.**
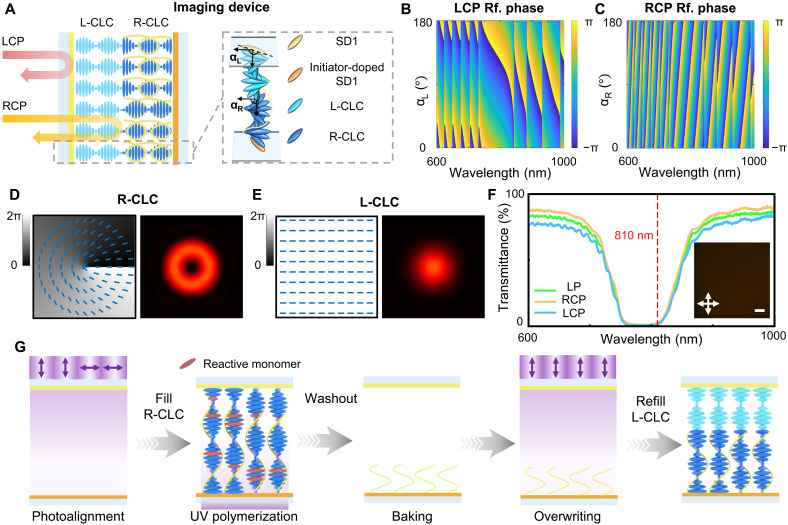
Design, characterization, and fabrication of the imaging device based on bichiral CLCs. (**A**) Schematic of the imaging device composed of bichiral CLCs. (**B** and **C**) Simulation results of the relative phase for the LCP and RCP light at different orientation angles and wavelengths. The color bars indicate the introduced phase’s value. (**D** and **E**) Theoretical distribution of the surface orientation and geometric phase and the corresponding simulated intensity profiles of the incident Gaussian beam for the R-CLC layer and L-CLC layer, respectively. (**F**) Experimental transmittance spectra for different polarized light. The inset is the micrograph of the imaging device. The white crossed arrows indicate the orthogonal polarizer and analyzer of the optical microscope. Scale bar, 200 μm. (**G**) Imaging device’s fabrication process based on the surface-initiated wash-out/refill process.

In the experiment, the bichiral CLC imaging device (Fig. 2A) was fabricated by a surface-initiated wash-out/refill process. The layer closer to the incident surface is the L-CLC, containing polymer-free LCs, and the R-CLC layer is stabilized by the polymer/LC nanocomposite. By means of the Berreman’s 4×4 matrix method (*49*), we can calculate the reflective phase modulation induced by L-CLC/R-CLC layers. The simulated results (Fig. 2, B and C) explicitly reveal how the reflective phase of different wavelength varies with the LC molecules’ orientation at the incident interfaces of the L-CLC/R-CLC layer (namely, α_L_ and α_R_; inset in Fig. 2A). On the basis of this phase modulation, we imprinted the spiral phase pattern (Fig. 2D) on the R-CLC chiral superstructures and the uniform orientation (Fig. 2E) on the L-CLC. Under ideal Gaussian beam incidence, opposite CLC layers exhibit donut-shaped (corresponding to edge image) and Gaussian (corresponding to bright-field image) intensity distributions, respectively. This confirms that the bichiral imaging device can perform two imaging modes dependent on the incident polarization. Figure 2G demonstrates the key procedures of sample fabrication (see more details in Materials and Methods). Figure 2F clearly displays the obtained PBGs encompassing the wavelength of 810 nm, together with its micrograph. The PBGs almost remain consistent under LCP, RCP, and LP incidence, which is consisted with the simulation results (Fig. 2, B and C). The micrograph exhibits a dark red coloration, conforming its PBG range.

**Fig. 3.**
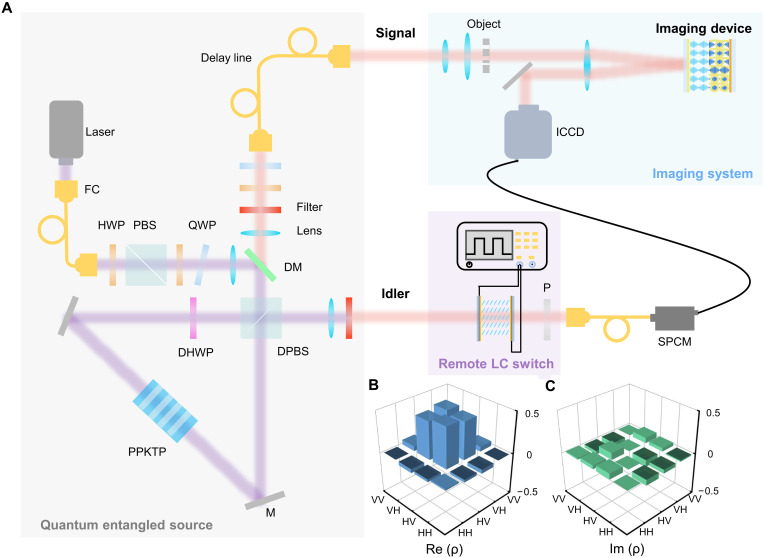
Optical setup of the entangled light source and its characterization. (**A**) Whole experimental setup including the quantum entangled source, imaging system, and remote LC switch. Abbreviations: FC, fiber coupler; HWP, half-wave plate; PBS, polarization beam splitter; QWP, quarter-wave plate; DM, dichromatic mirror; DHWP, dual-wavelength HWP; DPBS, dual-wavelength PBS; M, mirror; PPKTP, periodically poled KTiOPO_4_; P, polarizer; SPCM, single-photon counting module. (**B** and **C**) Real and imaginary parts of the density matrix of the output entangled photon pairs, which is reconstructed on the basis of quantum state tomography.

A schematic illustration of the experimental setup is presented in Fig. 3A, detailing the optical alignment and instrumentation arrangement. To generate polarization-entangled photon pairs, a frequency-degenerate type II quasi-phase-matched colinear spontaneous parametric downconversion (SPDC) process is used. A continuous-wave diode laser at 405 nm serves as the pump source. A 10-mm-long periodically poled KTiOPO_4_ (PPKTP) crystal (Raicol Crystals) with a 10-μm poling period is integrated into the polarization Sagnac interferometer and temperature stabilized at 27.8° ± 0.1°C using a stable thermoelectric controller. Besides, dual-wavelength antireflection coatings at 405 and 810 nm are applied to minimize the interface losses. The SPDC-generated photon pairs are collimated through achromatic doublet lenses with a focal length of 200 mm. Two 3-nm band-pass filters are placed before coupled into single-mode fibers to ensure frequency and spatial purity. To compensate for the time delay caused by the ICCD and capture the heralded image, a 20-m-length single-mode fiber is added in front of the imaging system. A half-wave plate (HWP) and a quarter-wave plate (QWP) are used to compensate for the polarization influence introduced by the optical fiber. Last, the output polarization-entangled state can reach the form of the aforementioned Bell state. The density matrix ρ of the experimental output is measured and reconstructed by means of quantum tomography (Fig. 3, B and C). The fidelity of our reconstructed state with respect to the pure Bell state $|\psi_{1}\rangle$ is defined as $F\left(|\psi_{1}\rangle ,\rho \right)=\langle \psi_{1}|\rho |\psi_{1}\rangle$, which is calculated as 92.7%.

In the imaging system, the signal photons pass through the beam expander and the target object positioned at the front focal plane of the lens (*f* = 150 mm). The center of the lens is slightly offset from the horizontal position of the incident beam. Combined with a bichiral CLC imaging device and a mirror, a reflective 4*f* system is constituted. On the basis of the shift-invariant property of the 4*f* system, the imaging device induced phase modulation in the spatial frequency domain can be performed in such an offset condition. Corresponding operations under different polarizer and analyzer configurations are exhibited in fig. S1, verifying two eigen polarization-dependent reflective channels. The resolution of the heralded imaging system is presented in fig. S2. On the other hand, the idler photons serve as an external trigger for the ICCD. Combined with wave plates and a polarizer, the desired polarization can be dynamically selected. Because of the polarization entanglement, the polarization of signal photons will be remotely switched under the coincidence measurement, and thus the imaging mode will be changed accordingly. This implies that imaging mode switching can be achieved without mechanical instrument contact, which can minimize the introduced influence.

### Electrically tunable remote switching of trimode quantum imaging

**Fig. 4.**
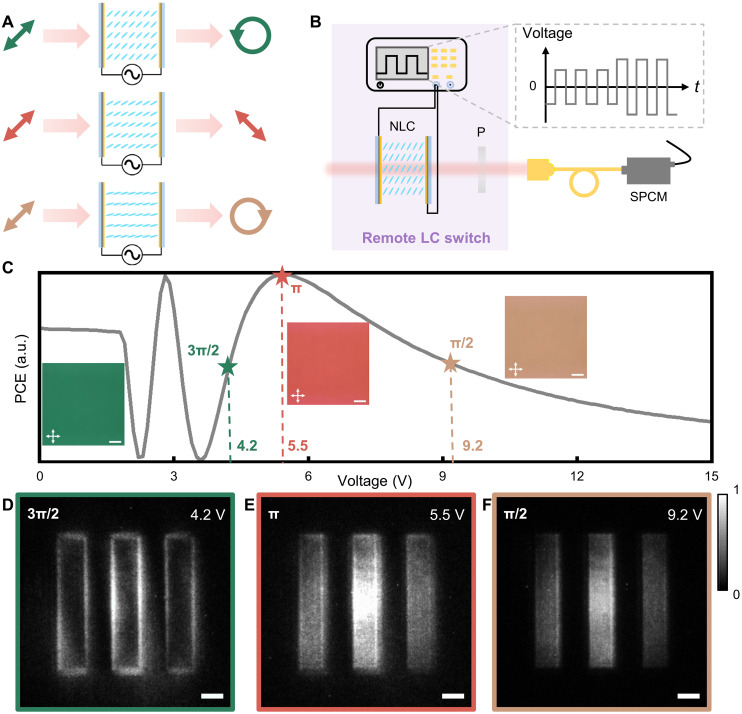
Electrically tunable effect of the NLC wave plate, the sketch of the remote NLC switch, and the corresponding quantum imaging results under different voltages. (**A**) Manipulation of light polarization introduced by the NLC wave plate under different voltages. (**B**) Sketch of the remote NLC switch controlled by an external 1-kHz alternating voltage. (**C**) PCE curves under different voltages. The insets show the micrographs of the NLC sample under the corresponding voltages, with phase retardation of 3π/2, π, and π/2, respectively. The white crossed arrows indicate the orthogonal polarizer and analyzer of the optical microscope. a.u., arbitrary units. Scale bars, 200 μm. (**D** to **F**) Output images corresponding to edge detection (D), superposition imaging (E), and bright-field imaging (F) are captured by the ICCD camera. Scale bars, 500 μm.

To realize the dynamic remote control, we introduce the NLC wave plate into the idler arm. Because of the electrically tunable effect, the tilted angle of NLC molecules can be adjusted by applying the external square-wave signal at 1 kHz. The phase retardation for 810 nm is modulated correspondingly, thereby altering the polarization state of output light (Fig. 4A). Figure 4C shows the relationship between the polarization conversion efficiency (PCE) and applied voltage of the NLC wave plate. The variation in PCE indicates a continuous change of the phase retardation. Here, three distinct conditions are selected in the experimental configuration. The corresponding micrographs of the NLC wave plate are shown in the insets of Fig. 4C, together with the respective phase retardations of 810 nm labeled. The color of micrographs depends on the wavelength satisfying the half-wave condition at each voltage.

For the remote LC switch, the NLC wave plate combined with a polarizer is inserted into the idler arm (Fig. 4B). The relative angle between them is 45°, that is, θ = 45° in Eq. 3, and the polarizer is used to select vertically polarized light, whereas the ΔΓ is dynamically adjusted by external applied voltage. Subsequently, the polarization components of idler photons are selected out as *R*, *V*, and *L* to trigger the ICCD, resulting in three imaging modes thanks to the polarization entanglement. Figure 4 (D to F) presents the experimental results with a remarkable SNR of edge detection, superposition imaging, and bright-field imaging corresponding to the three conditions above, respectively. Here, the bright-field image and edge image can reveal the morphology information and more accurate outline of the object, respectively, which exhibit the whole object roughly and further highlight the most featured information of target object to reduce the processed data amount. The superposition image specifically shows the overlap of the above two images, which can theoretically reveal the gradient direction if extracting the linear polarization component by another analyzer and has potentials in quantitative phase gradient imaging (*50*, *51*).

**Fig. 5.**
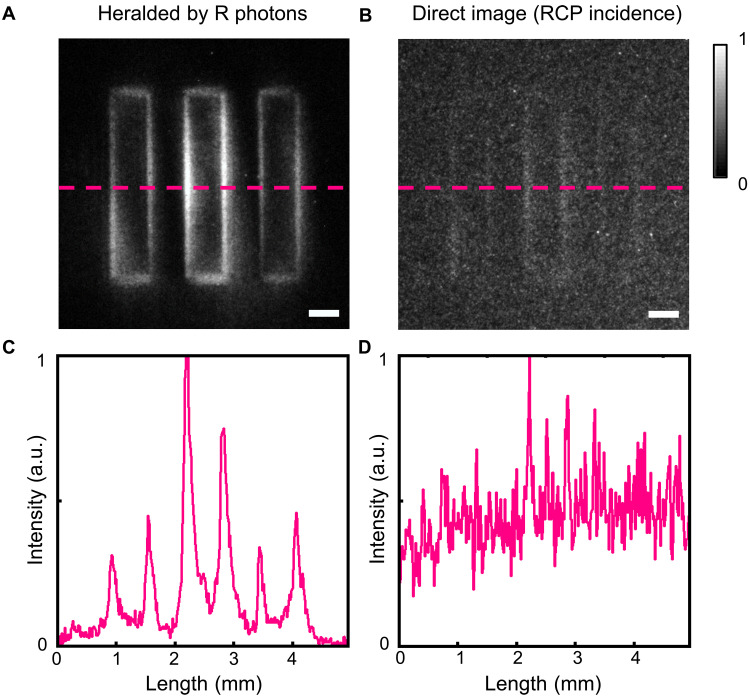
Comparison of heralded quantum imaging and direct imaging. (**A**) Edge image heralded by the R photons. (**B**) Direct image for R incidence. Scale bars, 500 μm. (**C** and **D**) The pink curves correspond to the relative intensities of dashed lines in (A) and (B).

In the experimental framework, the idler photon acts as a herald to activate the ICCD gate, ensuring temporal synchronization between the arrival of the signal photon and the ICCD capture. Therefore, the most ambient noise derived from the environment is efficiently prevented, and the image demonstrates an ultrahigh SNR based on the time correlation. To vividly verify its antinoise property, the heralded single-photon imaging and direct imaging without heralding are demonstrated for comparison [Fig. 5A (i.e., Fig. 4D) and Fig. 5B; see more comparisons in figs. S3 and S4]. The direct image is captured under continuous ICCD exposure with an exposure time equivalent to that used for the heralded acquisition, and the NLC wave plate is placed in front of the target to directly select out the incident polarization. Also, both illumination and environmental light remain unchanged except for whether the idler photons serve as the herald. Figure 5 (C and D) visually exhibits the high contrast enhancement enabled by the heralded imaging. Obviously, the SNR has been drastically improved compared to the direct image. The detailed calculation can be seen in Supplementary Text 2. In addition, the heralded single-photon imaging without remote switch is also presented in fig. S5, which exhibits a similar high SNR but lacks the nonlocality. In all, this proposed scheme can exhibit the coincidence image with a high quality and high contrast, which can be effectively used in photon-limited scenarios.

### Ultrafast remote control of quantum edge imaging

**Fig. 6.**
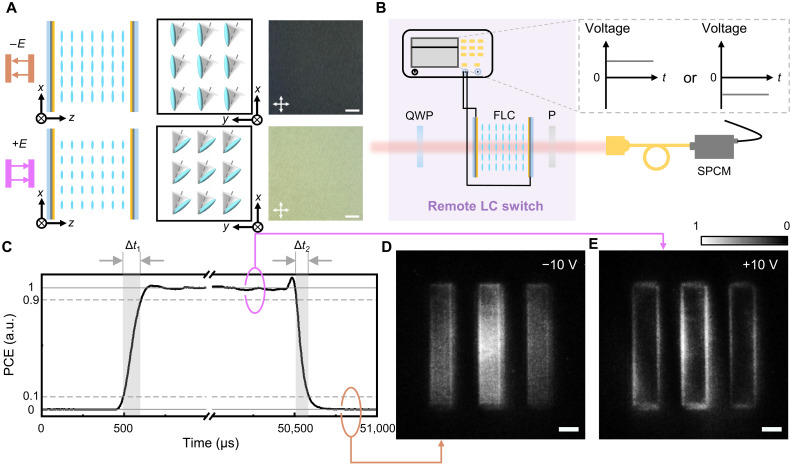
Ultrafast electro-optical effect of FLCs, the sketch of the remote FLC switch, and the corresponding quantum imaging results under an electric field of opposite polarities. (**A**) Scheme of molecule’s rotation and the corresponding micrographs. Scale bars, 200 μm. (**B**) Sketch of the remote FLC switch controlled by the external electric field. (**C**) Rising edge and falling edge of the experimental PCE curve indicate the ultrafast response time Δ*t*_1_ and Δ*t*_2_ of the FLC wave plate. (**D** and **E**) Output images corresponding to bright-field imaging and edge detection captured by the ICCD camera. Scale bars, 500 μm.

Furthermore, to achieve the ultrafast optical switch and improve the time efficiency of the quantum imaging system, an FLC wave plate is introduced. Without the external electric field, the FLC molecules are arranged averagely on the surface of the cone with a fixed tilt angle and compose a helical twisting lamellar structure. The electrically suppressed helix FLC mode is outstanding for the reconfigurable optical axis and ultrafast electro-optical response. For planarly aligned FLC superstructures with smectic layers perpendicular to the substrates, the helix will be suppressed by applying a direct voltage exceeding the threshold. The electric torque resulting from the interaction between spontaneous polarization of FLCs and external electric field switches the FLC director orientation. FLC molecules can rotate along the conical surface simultaneously and are realigned to one side of the cone. This transition is equivalent to the axis rotation of a wave plate. The rotation direction depends on the polarity of an applied electric field, and the differential rotation angle between positive and negative polarities is ~45° (Fig. 6A). Here, the brightness variation in micrographs also reveals a change in director orientation, indicating two operating modes in the remote FLC switch.

In the experiments, we fabricated an FLC wave plate with π phase retardation at 810 nm (see details in Materials and Methods) and then combined it with a QWP and a polarizer in the idler arm. The initial axis orientation of the FLC wave plate is 22.5°, whereas the QWP is placed along the 45° direction and the polarizer projects the output polarization state on ∣V〉. By switching the polarity of an external electric field (Fig. 6B), the θ of an equivalent wave plate in Eq. 3 with ΔΓ = π/2 is adjusted from −45° to 45°, leading to the polarization switching between the RCP and LCP. In particularly, the FLC wave plate exhibits marvelous ultrafast electro-optical response, enabling a submillisecond-scale switching between two quantum imaging modes. Figure 6C clearly exhibits the ultrashort switching time between the edge detection (Fig. 6E) and bright-field imaging (Fig. 6D). The response time are measured as Δ*t*_1_ = 108 μs and Δ*t*_2_ = 77 μs, respectively. In addition, it also demonstrates the glorious antinoise property, which is well verified by the comparison between Fig. 6 and fig. S6. This provides an innovative scheme for rapid remote control and offers a critical advantage for time-resolved applications and sequential logic control scenarios.

## DISCUSSION

In summary, the dynamic-tunable heralded single-photon imaging scheme is proposed and demonstrated on the basis of versatile LCs. The bichiral CLC is introduced to create the imaging device based on its spin-decoupled geometric phase manipulation, enabling the multifunctional quantum imaging. The NLC wave plate endows the capability of multimode switching due to its electrically tunable effect. The time correlation is optimized to reduce the ambient noise under coincidence measurement, and all results demonstrate a high SNR. Besides, to fulfill the high-speed switching, we introduce the FLC wave plate to nonlocally select the quantum imaging mode by alternating the polarity of an applied electric field. The quantum source is too weak to cause any irreversible damage to the detected samples, indicating the nondestructive nature of the detection. Benefiting from the quantum entanglement and LC’s stimuli-responsiveness, such active remote control harnesses the flexibility of heralding arm and ensures the high mechanical stability and optical convenience. This scheme exhibits remarkable imaging quality at an ultralow level of photon flux and can synchronously extract different morphological information of the object in photon-limited scenarios.

In addition, different liquid crystalline mesophases exhibit distinct advantages. The multidimensional light manipulation and rapid switching capabilities of various LCs have been well verified in quantum imaging. For the flipped bichiral CLC device, the captured heralded images always demonstrate the edge extraction for arbitrary incidence (fig. S7), which is consistent with the theoretical analysis. The function of LC devices shows a good robustness around room temperature, indicated by its stable transmittance spectra under different environment temperature (fig. S8). When it deviates substantially and even exceeds the phase transition point of LC materials, the refractive index and LC molecule arrangement will be notably affected, thus reducing the imaging quality. Overall, this LC-based heralded single-photon imaging scheme is reliable in daily real-world scenarios. Besides the used photoalignment technology, LC optical valves also provide an effective approach for generating optical vortices (*52*, *53*), which can be introduced to offer dynamic tunability and extend potential applications. Furthermore, an optical vortex array together with spiral phase metrology may facilitate abundant feature extractions (*54*, *55*). In the future, according to specific scenarios in the quantum technology, selecting appropriate liquid crystalline mesophases and methods could enable more versatile functionalities. In a word, this work discloses LC’s unprecedented capabilities in the on-demand manipulation of entangled photons and promotes an effective and dynamic platform for quantum information processing, intelligent recognition, and biomicroscopy.

## MATERIALS AND METHODS

### Materials

For bichiral CLCs, the photoinitiator-doped SD1 solution was prepared by combining the SD1 solution with 0.15 wt % diphenyl ketone. Upon ultraviolet (UV) exposure, SD1 molecules reoriented perpendicular to the UV linear polarization, thus directing the LC molecules to align parallelly. The initial R-CLC mixture consisted of NLC E7 (HCCH, China), right-handed chiral dopant R5011 (HCCH, China), and LC reactive monomer RM257 (HCCH, China) at a weight ratio of 78.7:1.3:20. The central wavelength of the PBG in this initial R-CLC was 1135 nm. The refilled L-CLC consisted of NLC E7 and left-handed chiral dopant S5011 (HCCH, China) at a weight ratio of 97.62:2.38, with its PBG covering 810 nm. For the NLC wave plate, NLC E7 (HCCH, China) was chosen. For the FLC wave plate, the sulfonic azo-dye SD1 was dissolved in dimethylformamide at a concentration of 0.35 wt %. The FLC material (BEAM Co., USA) with the helix pitch *P*_0_ = 245 nm, spontaneous polarization *P*_S_ = 110 nC/cm^2^, and tilt angle θ = 25° near ideal 22.5° was chosen, with a phase transition of isotropic → SmA* → SmC* at 82° and 72°C, respectively.

### Sample fabrication

For the bichiral CLC device, after UV-ozone treatment, the indium-tin-oxide (ITO) glass substrates were spin-coated with either SD1 solution or photoinitiator-doped SD1 solution and cured at 100°C for 10 min to form an alignment layer. Then, a photoinitiator-free substrate was assembled with a photoinitiator-doped substrate. A 15-μm spacer/glue mixture was applied along the two edges to form an empty LC cell. Then, a surface-initiated wash-out/refill process was implemented (Fig. 2G). First, a multistep partly overlapping exposure process was conducted using a digital micromirror device (DMD)–based photopatterning system. This process achieved the patterned alignment of the SD1 layer with a spiral pattern (*q*-plate, *q* = 0.5). After that, the R-CLC mixture was filled into the cell at 85°C. During polymerization, the cell was exposed to nonpolarized UV light (365 nm, 4.6 mW/cm^2^) from the photoinitiator-doped side for 15 min. Subsequently, the cell was immersed in acetone for 12 hours to remove the unreacted molecules, leaving behind a shrunk polymer scaffold that maintained the initial alignment and chirality. To ensure that the final bichiral CLC operates correctly on the wavelength of the polarization-entangled source (810 nm), a baking process at 120°C for 7 min is required. Generally, the higher temperature will reduce the polymer scaffold’s rebound ability, resulting in a blue shift for R-CLCs in the final PBG. Afterward, the SD1 alignment on the photoinitiator-free substrate is rewritten by the second exposure to uniformly polarized UV light for 10 min to form a uniform alignment for the refilled LCs. Last, L-CLCs are refilled to form the bichiral CLC quantum imaging device.

For the NLC wave plate, the LC cell with a thickness of 9.5 μm was uniformly photopatterned and then filled with NLC E7. For the FLC wave plate, two bare ITO-coated glass substrates (1.5 cm by 2.0 cm) were prepared. Only one substrate underwent UV-ozone treatment and then was spin-coated with the photoalignment material, followed by thermal curing at 100°C for 10 min. Two substrates were separated by spacers and sealed with the UV glue to form a 1.4-μm-thick cell. After polarized UV exposure (405 nm, 21.78 mW/cm^2^) for 10 min, the empty cell was imprinted with a uniform alignment. The FLC material was filled into the cell at 85°C and then gradually cooled from the isotropic phase to the SmC* phase. The cooling rate was 0.1°C/min when approaching the phase transition points within the range of ±2°C. At other times, the cooling rate was 1°C/min.

### Information for the ICCD

The used ICCD camera (Andor iStar DH334T-18 U-73) has an effective pixel count of 1024 * 1024 with a pixel size of 13 μm * 13 μm. During the running time, it is air-cooled into −30°C with 20% quantum efficiency. To capture heralded single-photon images, the ICCD camera operates an external trigger mode and a digital delay generator (DDG) gate mode. The corresponding measurement conditions are 10-s discrete exposure of each frame (*T*_ex_), 50 accumulative times (frame number, *N*), 3600 gain, and 6-ns gate width (*T*_w_) with a delay time of 7 ns. For the direct imaging, the ICCD camera operates an internal trigger mode and a fire-only gate mode. The exposure time is set at 1.8 s with 3600 gain and is equivalent to the heralded imaging condition, which follows *T*_in_ = *N* * *T*_ex_ * *T*_w_ * *C*. *C* is the count per second captured in the heralding arm, ~6 × 10^5^/s. All images captured by the ICCD have been normalized respectively.
