# Supplementary material for: Advancing quantum imaging: Electrical tunability enabled by versatile liquid crystals
Source: Sci Adv. 2026 Jan 28;12(5):eadz8962. doi: 10.1126/sciadv.adz8962 (PMC12851020; doi:10.1126/sciadv.adz8962)
Supplement: Supplementary file 1 — Supplementary Text 1 and 2 Figs. S1 to S8 [file sciadv.adz8962_sm.pdf]

Science Advances
AAAS

Supplementary Materials for

Advancing quantum imaging: Electrical tunability enabled by versatile liquid crystals

Dong Zhu *et al.*

Corresponding author: Zhi-Xiang Li, zxli@nju.edu.cn; Peng Chen, chenpeng@nju.edu.cn; Yan-Qing Lu, yqlu@nju.edu.cn

Sci. Adv. **12**, eadz8962 (2026)
DOI: 10.1126/sciadv.adz8962

This PDF file includes:

Supplementary Text 1 and 2
Figs. S1 to S8

Supplementary Text 1.

Principle of spin-decoupled geometric phase modulation in bi-chiral CLCs

The arbitrary polarization light can be expressed as the superposition of LCP and RCP components, as the following form:

$$\left|E\right\rangle_1 = A\left|L\right\rangle + Be^{i\Delta\psi}\left|R\right\rangle. \quad \text{(S1)}$$

Here, the parameters A and B are real numbers which satisfy the normalization condition. $\Delta\psi$ is the initial phase difference between two opposite circularly polarized components. $\left|L\right\rangle$ and $\left|R\right\rangle$ represent LCP and RCP, respectively. Because of this spin-decoupled geometric phase introduced by the bi-chiral CLCs, the reflective output state can be expressed as

$$\left|E\right\rangle_2 = \alpha e^{i\Phi_\mathrm{L}}\left|L\right\rangle + \beta e^{i\Delta\psi}e^{i\Phi_\mathrm{R}}\left|R\right\rangle. \quad \text{(S2)}$$

Both of the reflective phase Φ_L and Φ_R are composed of the geometric phase and the propagation phase, following:

$$\Phi_\mathrm{L} = \Phi_\mathrm{L,G} + \Phi_\mathrm{L,P} \text{ and } \Phi_\mathrm{R} = \Phi_\mathrm{R,G} + \Phi_\mathrm{R,P}. \quad \text{(S3)}$$

Here, $\Phi_\mathrm{L,P}$ and $\Phi_\mathrm{R,P}$ refer to the propagation phases derived by the reflected LCP and RCP light, respectively. $\Phi_\mathrm{L,G}$ and $\Phi_\mathrm{R,G}$ refer to the geometric phases for LCP and RCP light.

Due to the spin-selected Bragg reflection characteristic of the CLC, incident light is reflected close to the surface of the corresponding-handed CLC layer. Consequently, the propagation phase can be expressed as

$$\Phi_\mathrm{L,P} = \Phi_\mathrm{L,0} \text{ and } \Phi_\mathrm{R,P} = \Phi_\mathrm{R,0} + \frac{4\pi}{\lambda}n_\mathrm{eff}d, \quad \text{(S4)}$$

where the $\Phi_\mathrm{L,0}$ and $\Phi_\mathrm{R,0}$ refer to the extra phases induced during the Bragg reflection of LCP and RCP, and can be calculated by the exact solution at normal incidence. n_eff is the effective refractive index of L-CLC layer for the transmitted RCP light component, d is the thickness of the active L-CLC layer. While the exact expression of n_eff can be derived by solving the wave equation of normal modes, a common approximation practical simplification, $n_\mathrm{eff} \approx \sqrt{\left(n_\mathrm{e}^2 + n_\mathrm{o}^2\right)/2}$, is to use the average of n_e and n_o. Both of these propagation phases are spatially-invariant and only result in a constant phase difference between the LCP and RCP component.

In the other hand, the geometric phase $\Phi_\mathrm{L,G}$ and $\Phi_\mathrm{R,G}$ are induced owing to the spin-orbit interaction, depending on the spatially-variant CLC's director orientation on the incident side respectively, following:

$$\Phi_\mathrm{L,G} = -2\alpha_\mathrm{L} \text{ and } \Phi_\mathrm{R,G} = 2\alpha_\mathrm{R}. \quad \text{(S5)}$$

α_L and α_R are mutually independent, and have been shown in Fig. 2A, which can be encoded by photopatterning in sequence. Thanks to the spin-decoupled geometric phase, two arbitrary phase modulations can be imposed on the incident light. Furthermore, for LP light incident, the output result will be superposition of LCP and RCP light components with the corresponding phase modulation. The specific value of introduced reflective phase can be calculated by Berreman's 4×4 matrix method, as shown in Fig. 2 (B and C).

.

Supplementary Text 2.

Noise analysis

In order to analyze the SNR of captured images, we should first explain the principle of the denoising property introduced by heralded imaging. The quantum entangled source can generate one pair of photons through the SPDC process, because the interaction strength is small and the probability of generating more than one pair is negligible. The signal and idler photons within a single entangled photon pair exhibit a pronounced time correlation. It ensures that the probability of signal photons captured by ICCD camera is much larger than the probability of noise photon when the heralded photon arrives.

To evaluate the quality of our imaging system, the SNR is calculated as follows:

$$SNR = 20\log_{10}\left(\frac{\sqrt{\sum_{j}^{n} t_j^2}}{\sqrt{\sum_{j}^{n}\left(\frac{m_j}{s} - t_j\right)^2}}\right). \quad \text{(S6)}$$

m (t) denotes the measured experimental (target ideal) image and *j* denotes the pixel index ranging from 1 to *n*. *s* is used to renormalize the experimental image. The ideal edge image is calculated with the same size as the experimental ones. We calculate the SNR for Fig. 5 (A and B), as 4.39 dB and -16.43 dB. It demonstrates the appealing anti-noise property from heralded imaging.

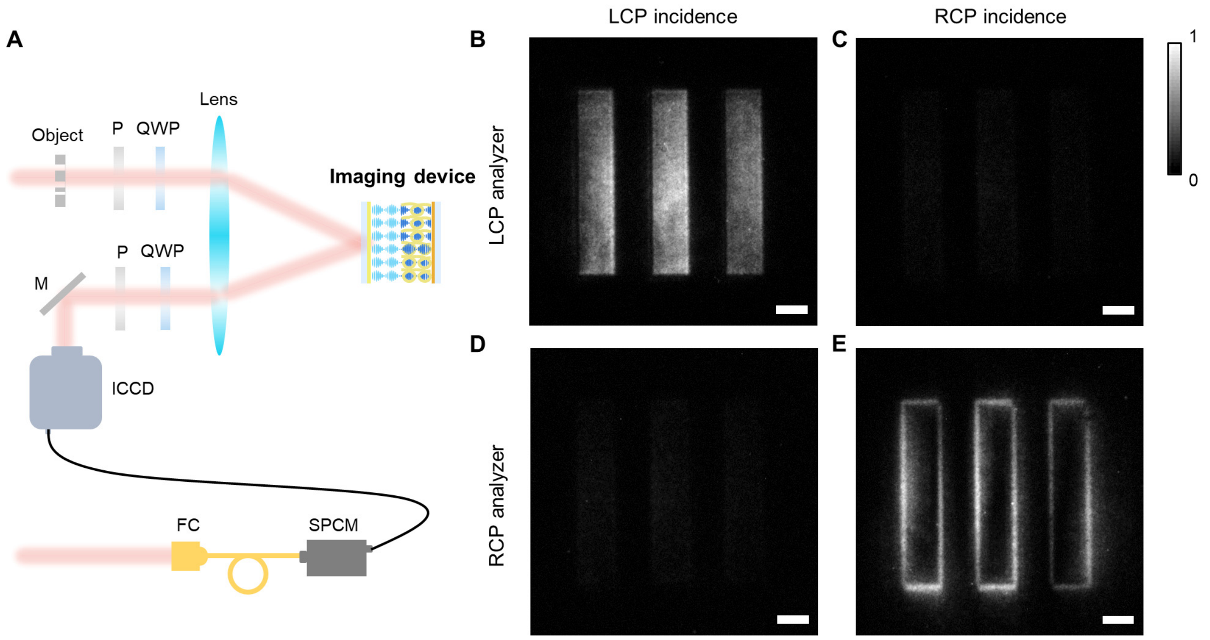

Fig. S1.

Heralded single photon imaging under different polarization configurations. (**A**) The experimental setup to capture the image under different circular polarizer and analyzer configurations. (**B to E**) The corresponding images captured by four combinations of circular polarizer and analyzer. The intensities of the four images are normalized uniformly. The scale bars are 500 μm.

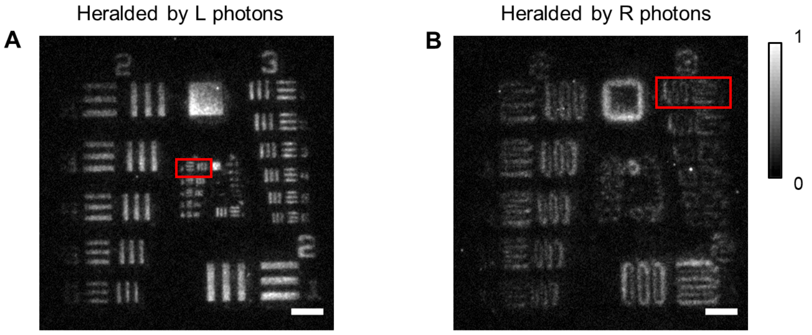

Fig. S2.

The resolution of the heralded single photon imaging. (**A**) The heralded images of resolution target corresponding to bright-field imaging and (**B**) edge detection exhibit the resolution of the imaging system. The line pairs in the red box demonstrate the resolution in these imaging modes, respectively. The scale bars are 500 μm.

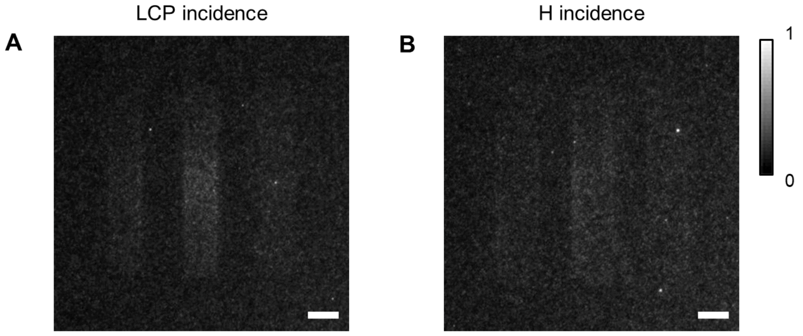

Fig. S3.

The direct images selected by NLC wave plate. (**A**) Direct images correspond to bright-field imaging for LCP incidence and (**B**) superposition imaging for horizontal polarization (H) incidence captured by ICCD camera, respectively. The scale bars are 500 μm.

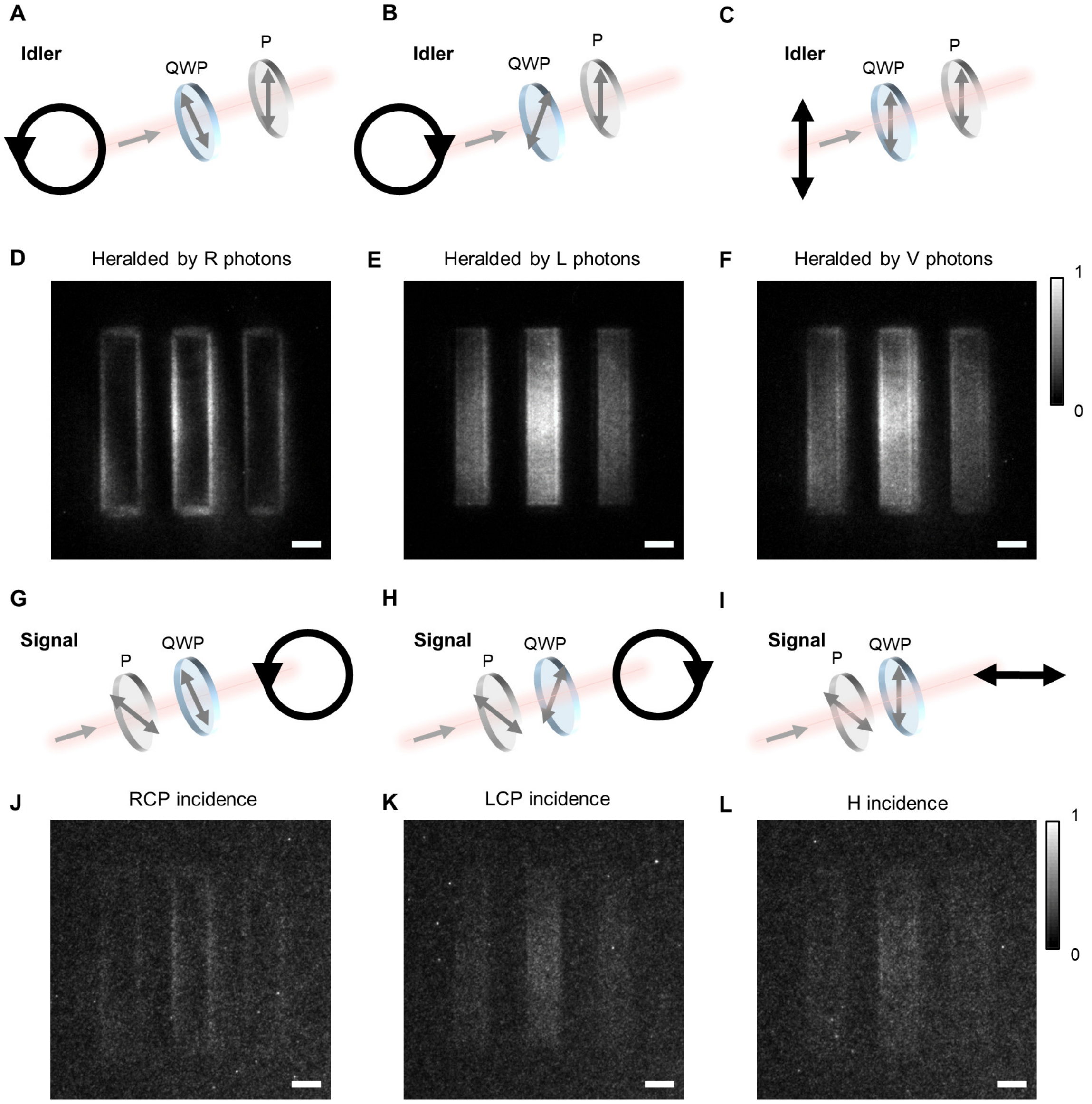

Fig. S4.

The heralded images and direct images selected by a QWP. (**A** to **C**) The polarization of heralding photons is selected by a QWP and a polarizer and projected on RCP, LCP and vertical polarization (V) state. (**D** to **F**) The heralded images corresponding to (A to C) exhibit the edge detection, superposition imaging and bright-field imaging, respectively. (**G** to **I**) The polarization of incident photons is selected by a QWP and a polarizer projected on RCP, LCP and H state. (**J** to **L**) The direct images corresponding to (G to I) exhibit the edge detection, superposition imaging and bright-field imaging, respectively. The scale bars are 500 μm.

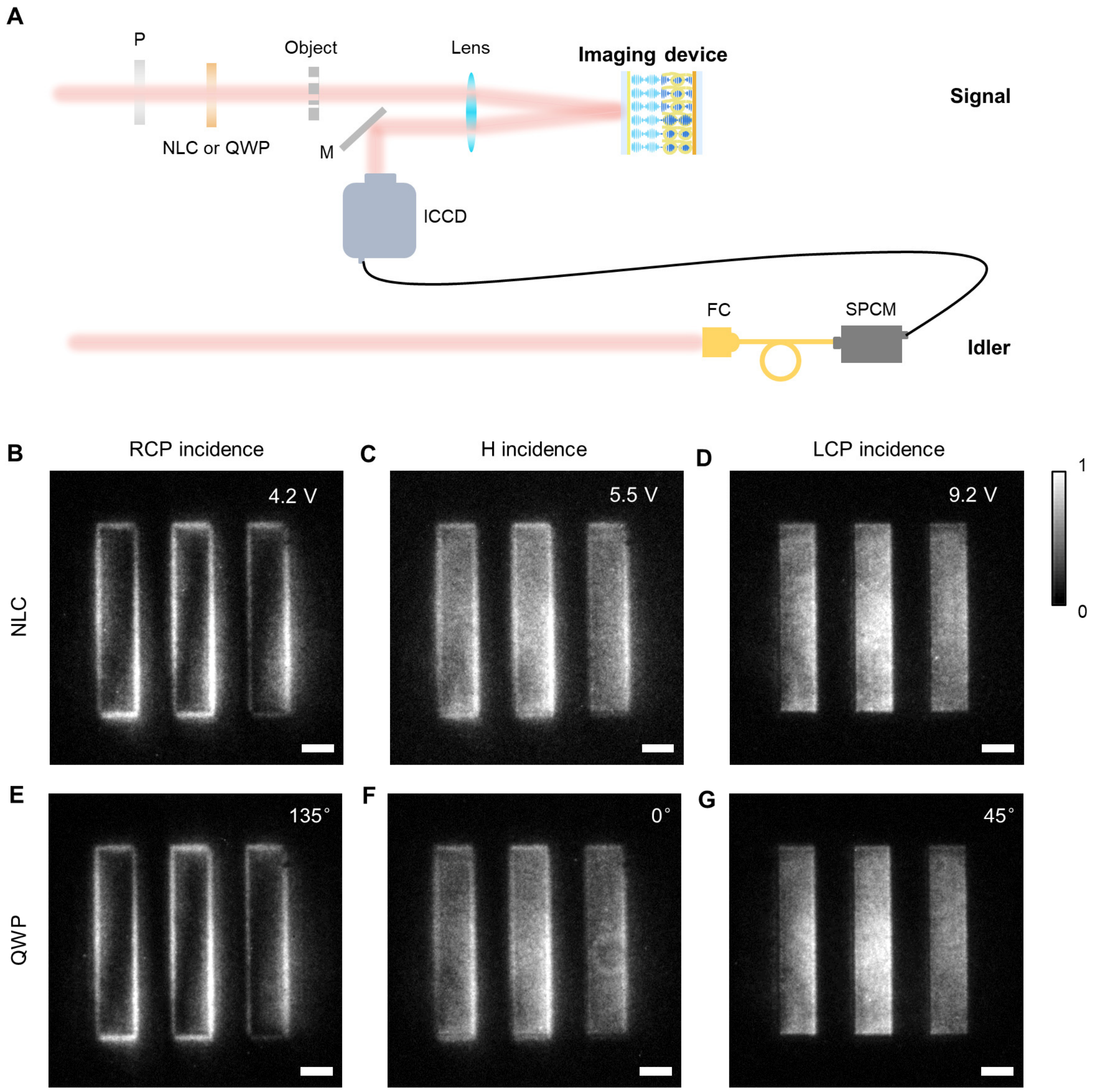

Fig. S5.

Heralded single photon imaging without remote switch. (**A**) The experimental setup to realize the heralded single photon imaging without remote switch. (**B to D**) The output images for RCP incidence (B), H incidence (C) and LCP incidence (D) based on NLC wave plate. (**E to G**) The output images corresponding to RCP incidence (E), H incidence (F) and LCP incidence (G) based on QWP. The scale bars are 500 μm.

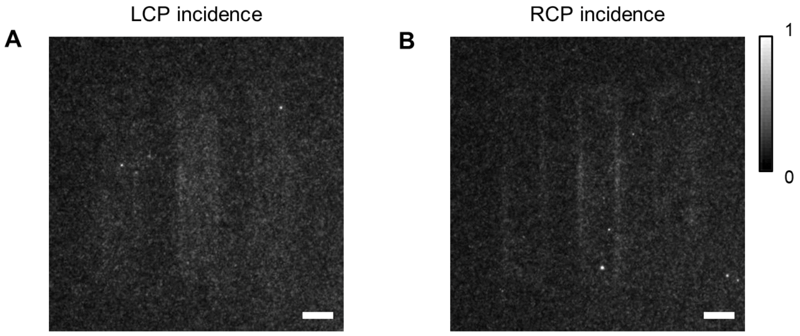

Fig. S6.

The direct images selected by FLC wave plate. (**A**) Direct images correspond to bright-field imaging for LCP incidence and (**B**) edge detection for RCP incidence captured by ICCD camera, respectively. The scale bars are 500 μm.

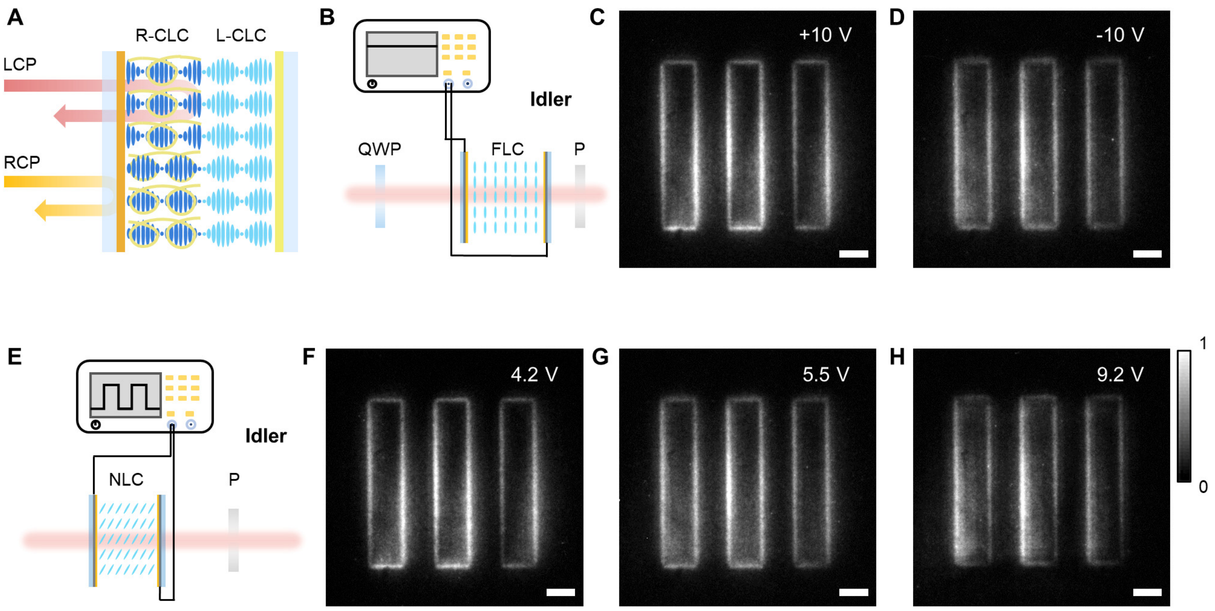

Fig. S7.

The heralded quantum images with the flipped imaging device, namely, incident from the R-CLC layer. (**A**) Schematic of the imaging device composed of flipped bi-chiral CLCs. (**B**) The sketch of remote FLC switch controlled by the external electric field. (**C** and **D**) Output images corresponding to different circular polarization selection captured by ICCD camera. (**E**) The sketch of remote NLC switch controlled by external 1 kHz alternating voltage. (**F** to **H**) Output images corresponding to nonlocally RCP, V and LCP state selection captured by ICCD camera. The scale bars are 500 μm.

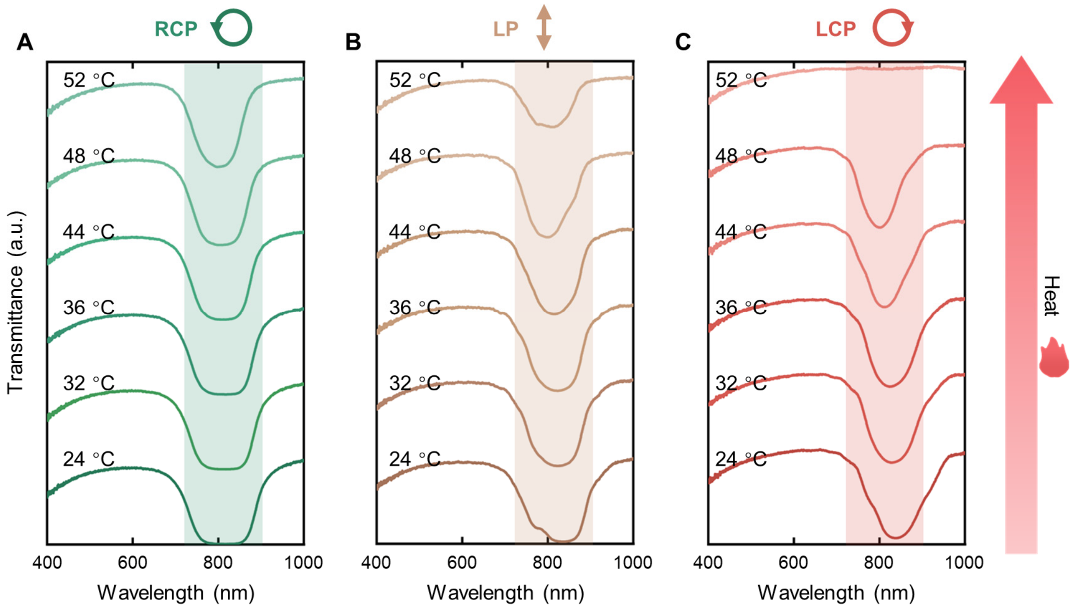

Fig. S8.

Temperature-dependent evolution of the transmittance spectra of the bi-chiral CLCs. (**A** to **C**) Transmittance spectra under illumination with RCP (A), LP (B), and LCP (C) light at different temperatures. The shaded areas mark the reflection band.
